# The Action of Naphthaline on the Visual Organs

**Published:** 1888-03

**Authors:** 


### THE ACTION OF NAPHTHALINE ON THE VISUAL ORGANS.

According to some recent experiments the ingestion of napthaline produces marked lesions of the retina, the optic nerve, the vitreous humor, and the crystalline lens, in guinea pigs. On the retina its administration is followed by small patches of a brilliant white, which rapidly increase in size and coalesce, principally in the neighborhood of the optic nerve. In large doses the retina is rapidly destroyed. In the vitreous humor a number of opalescent opacities appear, not unlike cholesterin. The most curious effects are those observable in the crystalline lens ; the appearance of the white spots is preceded by shaded lines, followed by the formation of opacities either on the posterior surface or round the edge immediately beneath the capsule. In a few days the lens will have become quite opaque. The histological examination shows that these patches are due to an exudation of round cells beneath the capsule and between the peripheral fibres of the lens.—*Medical Press and Circular.*

